# Supplementary material for: Comparative multiomics analysis of cell physiological state after culture in a basket bioreactor
Source: Sci Rep. 2022 Nov 23;12:20161. doi: 10.1038/s41598-022-24687-4 (PMC9686226; doi:10.1038/s41598-022-24687-4)
Supplement: Supplementary file 1 — Supplementary Information 1. [file 41598_2022_24687_MOESM1_ESM.zip › raw data/Proteomic raw data/6.KEGGenrichment/EG.vs.CG/DOWN/EG.vs.CG.KEGG_Enrich.scatterplot.pdf]

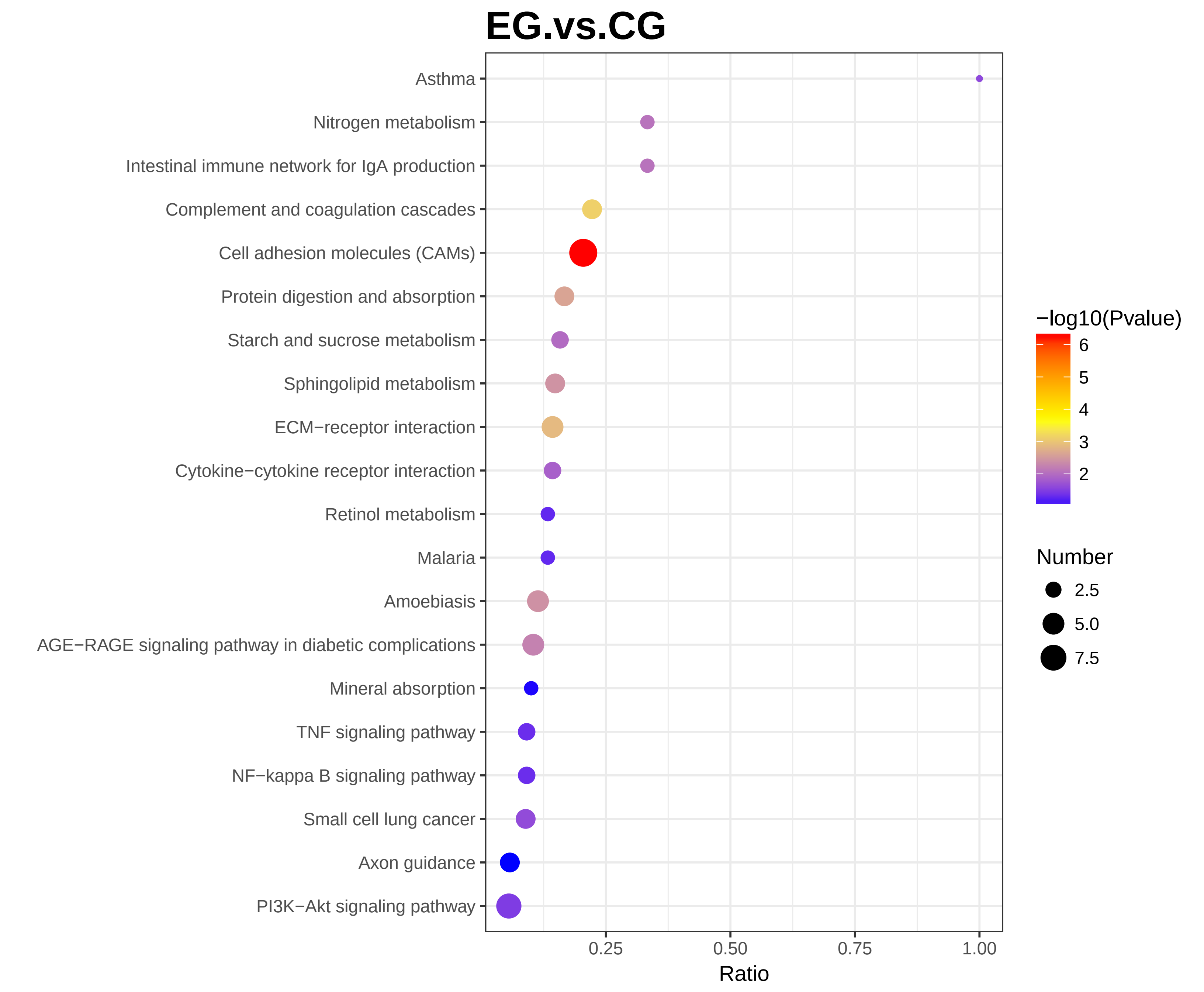
EG.vs.CG
Asthma
Nitrogen metabolism
Intestinal immune network for IgA production
Complement and coagulation cascades
Cell adhesion molecules (CAMs)
Protein digestion and absorption
Starch and sucrose metabolism
Sphingolipid metabolism
ECM−receptor interaction
Cytokine−cytokine receptor interaction
Retinol metabolism
Malaria
Amoebiasis
AGE−RAGE signaling pathway in diabetic complications
Mineral absorption
TNF signaling pathway
NF−kappa B signaling pathway
Small cell lung cancer
Axon guidance
PI3K−Akt signaling pathway
0.25
0.50
0.75
1.00
Ratio
−log10(Pvalue)
6
5
4
3
2
Number
2.5
5.0
7.5
